# A Hospitalist-Run Procedure Service Safely Streamlines Inpatient Intrathecal Chemotherapy

**DOI:** 10.7759/cureus.105448

**Published:** 2026-03-18

**Authors:** Ghadi Ghanem, Allyson Malone, Lillian Chen, Gary S Feigenbaum, Alexandra M Glaeser

**Affiliations:** 1 Department of Medicine, University of California, Los Angeles David Geffen School of Medicine, Los Angeles, USA; 2 Department of Medicine Statistics Core, University of California, Los Angeles David Geffen School of Medicine, Los Angeles, USA

**Keywords:** hospitalist medicine, intrathecal chemotherapy, intrathecal infusion, length of stay, lumbar puncture (lp), medical procedure, medical procedures, procedure service, thrombocytopenia, transfusion threshold

## Abstract

Background

In patients with hematologic malignancies, intrathecal chemotherapy by means of a lumbar puncture (LP) has found a critical role in delivering vital medications to patients with central nervous system (CNS) spread. However, many oncology patients who require this procedure have refractory thrombocytopenia that can lead to delays in intrathecal chemotherapy administration, as well as potentially the excess use of platelet transfusions, which are a limited resource. At our institution, intrathecal chemotherapy is delivered by radiology with a strict minimum number of platelets or a hospitalist-led medical procedure service (MPS), with a more individualized threshold. We sought to compare outcomes and complications of intrathecal chemotherapy administered using radiologist-guided fluoroscopy or bedside with the MPS.

Methods

We conducted a retrospective cohort study of adult inpatients referred for intrathecal chemotherapy from August 2019 to May 2022. An electronic medical record tool extracted demographics, laboratory results, and procedure timing. Relevant imaging within seven days of the LP was individually reviewed to determine major complications. Separately, patients were evaluated for traumatic taps based on their cerebrospinal fluid (CSF) red blood cell (RBC) count.

Results

There were 564 procedures that fit the inclusion criteria, 485 done by radiology and 79 by MPS. MPS teams conducted the procedure at lower platelet thresholds (49% with platelets below 50,000 in MPS as opposed to 6.4%, p < 0.001). Both teams had a low complication rate, 1.3% for MPS and 1.6% for radiology, though the MPS completed LPs sooner after consultation requests (six versus 24 hours; odds ratio {OR}: 0.41; p<0.001), with a 71% decrease in traumatic tap occurrence compared to those performed by radiology (OR = 0.29; p = 0.002).

Conclusions

Our study suggests that a hospitalist-run MPS can substantially expedite patient care in this vulnerable oncology population with a low complication rate, despite conducting the procedures with a higher proportion of patients with thrombocytopenia. Further research is warranted to determine MPS's impact on the length of stay (LOS), as well as potential cost savings associated with the procedure.

## Introduction

Lumbar punctures (LPs) are routinely performed procedures in the hospital setting, serving as an essential diagnostic tool for many neurologic diseases. This procedure also has utility as a therapeutic tool, particularly in the field of oncology, through the administration of intrathecal chemotherapy. This method allows for the direct delivery of chemotherapy drugs into the cerebrospinal fluid (CSF), bypassing the blood-brain barrier, which has been shown to reduce the incidence of the central nervous system (CNS) involvement of some cancers [1]. While LPs are generally safe and well-tolerated, serious complications are quite rare, with up to 0.3% of patients requiring a blood patch and 0.7% requiring hospitalization [2]. Intrathecal chemotherapy also carries further risk, including chemical aseptic meningitis, which can occur in up to 40% of patients [3]. Further serious complications can include leukoencephalopathy and myelopathy, both of which can appear as T2 hyperintensity on MRI [4,5].

Traditionally, internal medicine physicians performed the majority of LPs on patients admitted to medicine services. However, over the past decades, there has been a shift toward fluoroscopy-guided LPs by the radiology department in many areas of the country. There are likely multiple reasons for this, including the removal of LP training requirements in internal medicine residency programs by the Accreditation Council for Graduate Medical Education, increasing technical difficulty due to higher rates of obesity and an aging population, and escalating time constraints on physicians [6]. Nationwide, fewer internal medicine physicians are performing LPs [7]. In contrast, radiology has experienced an expansion in the variety and volume of minimally invasive procedures. Due to heightened demand for minimally invasive procedures, wait times for this specialized service can be long, which can introduce delays in care. In response to these factors, many academic medical programs have developed medical procedure services (MPS) to cohort a select group of internal medicine physicians, usually hospitalists, who are skilled at bedside procedures. We therefore sought to explore the safety and efficiency of MPS teams as compared to radiology in performing LPs and delivering intrathecal chemotherapy for admitted patients who require this. At our institution, the MPS has shown promise in expediting care for patients requiring central lines for chimeric antigen receptor T-cell therapy [8]. Furthermore, MPS promote more efficient care by decreasing the resources required to complete a procedure and helping to offload some of radiology's heavy workload. Patients can also avoid exposure to radiation during LPs performed by MPS with relatively high LP success rates of 81%-84%, with minimal complications [9,10].

Historically, there has been increased concern for the development of spinal hematomas in patients who undergo an LP in the setting of coagulopathies or who are receiving anticoagulation [11]. However, the impact of thrombocytopenia on bleeding in the setting of LP has been heavily debated. Current guidelines from the American Association of Blood Banks have a weak recommendation for prophylactic platelet transfusion in patients undergoing elective diagnostic LP with a platelet count of less than 50,000 cells/µL. However, this is based on very low-quality evidence [12]. A platelet threshold above the minimum required for safety has transfusion-related risks, including fever, anaphylaxis, infection, alloimmunization, and transfusion-related acute lung injury [13]. This is especially relevant in patients with refractory thrombocytopenia who require multiple transfusions, such as those with hematologic malignancies. Platelets are also a finite resource with frequent shortages in part due to donation patterns and a short shelf life, requiring thoughtful resource allocation [14-17]. Patients receiving intrathecal chemotherapy have a high frequency of thrombocytopenia due to the marrow-suppressive effects of active chemotherapy and hematologic malignancies. Prior studies have suggested that LPs may be completed safely at a platelet threshold lower than 50,000 cells/µL in this population [18]. Our MPS often performs LPs when platelets are less than 50,000 cells/µL, while our radiology department typically requires a strict platelet goal of 50,000 cells/µL.

Our investigation primarily sought to compare time to completion and complication rates between intrathecal chemotherapy performed using fluoroscopy with radiology or bedside with MPS teams utilizing ultrasound. Secondary outcomes from our study included the number of platelet transfusions and the chance of traumatic tap.

## Materials and methods

Study setting and design

We conducted a retrospective cohort study on databases that were obtained at a university-based academic medical center. All adult inpatients who were referred to the MPS or radiology for intrathecal chemotherapy between August 2019 and May 2022 were included in the analysis. The study was reviewed and determined to be in the exempt category by the university's institutional review (University of California, Los Angeles {UCLA} Institutional Review Board {IRB} number 23-001664).

Patient allocation

Patients were referred to MPS teams or radiology at the discretion of the primary inpatient team. The referral was conducted by means of placing an order within our electronic medical record for "consult to proceduralist" or "consult to radiology." Primary teams were also provided the option of paging either team for more expeditious requests. Due to workflow limitations, no randomization of patients was conducted.

Data collection and analysis

An electronic medical record search tool was created to extract variables in each patient's chart to include service date, sex assigned at birth, body mass index (BMI), international normalized ratio (INR), platelet count immediately preceding procedure, units of platelets transfused within 48 hours prior to the procedure, and red blood cell (RBC) count in CSF from the procedure. Wait times were calculated using the difference in hours from the order time to the completion of the procedure. Researchers manually examined patient charts and reviewed all imaging obtained within one week of each LP. Complications were considered as objective imaging findings of spinal epidural or subdural hematoma, cerebral herniation, or abscesses on imaging within one week of the procedure. Two blinded attending physicians subsequently independently reviewed the imaging to determine if the findings could reasonably be attributed to the intrathecal chemotherapy infusion or LP. The attendings were not made aware of the procedural details but did have access to subsequent documentation in order to determine if intervention was required (such as a blood patch). Traumatic taps were defined as a CSF RBC greater than 500 RBCs/mm^3^, a common definition in the literature [19]. This was also selected as past research has indicated that less than 500 RBCs/mm^3^ in collected CSF is associated with a 100% negative predictive value for subarachnoid hemorrhage and therefore is already used as a screening tool for practitioners [20].

Statistical analysis

We employed various regression methods to investigate potential relationships between key clinical outcomes of interest with exposure to the physician treating team (MPS or radiology) and platelet count. For continuous and binary outcomes, generalized estimating equations (GEEs) were employed using an exchangeable correlation structure and either an identity or binomial link function. Continuous outcomes were log-transformed prior to analysis. Count outcomes were modeled using zero-inflated negative binomial regression models, specifying the zero-inflation component as an intercept-only model. Robust standard errors were reported to handle clustering at the patient level.

Baseline characteristics were described by medians with interquartile ranges for continuous variables and n (%) for categorical variables. Wilcoxon rank sum tests were performed to evaluate differences between physician teams across continuous variables. Either Pearson's chi-squared test or Fisher's exact test (if any expected cell count was less than five) was performed to assess differences between physician teams across categorical variables. Statistical significance was set at α = 0.05. All analyses were performed in R version 4.4.2 (R Foundation for Statistical Computing, Vienna, Austria).

## Results

**Figure 1:**
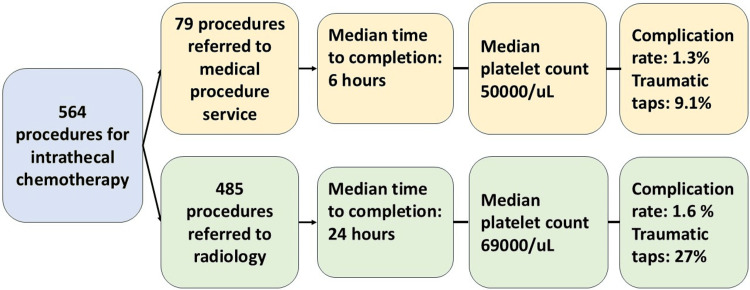
Study flow and outcome summary.

A total of 564 procedures fit the inclusion criteria. Four hundred eighty-five LPs were performed by radiology, and 79 by the MPS (Figure 1). In accounting for patients with recurrent procedures, there were a total of 188 unique patients in the radiology cohort, while there were 40 separate patients in the MPS group. Four patients who initially underwent the procedure with MPS teams had their procedure aborted and required radiology to complete the procedure. On average, patients who underwent procedures with radiology had two visits, while those in MPS had one (p = 0.077). Baseline patient characteristics, including age, biological sex, and body mass index, were similar between the groups (Table 1 and Appendices). For the purposes of data analysis, we considered each episode of intrathecal chemotherapy as an independent event. Outcomes clustered by individual patient are available in the Appendices.

**Table 1:** Patient characteristics and sequelae for individual lumbar punctures with intrathecal chemotherapy stratified by performing group: radiology or medical procedure service (MPS). ^1^Median (Q1 and Q3); n (%). ^2^Wilcoxon rank sum test; Fisher's exact test; Pearson's chi-squared test.

Variable	Radiology (N = 485)^1^	Medical Procedure Service (N = 79)^1^	P-value^2^
Body Mass Index	27 (23 and 31)	27 (24 and 32)	0.296
Missing	38	6	
Cerebrospinal Fluid Red Blood Cell Count	43 (3 and 1,000)	6 (1 and 61)	<0.001
Missing	16	2	
International Normalized Ratio (INR)	1.10 (1.10 and 1.20)	1.10 (1.00 and 1.10)	<0.001
Missing	8	2	
INR, Dichotomized			0.232
At Least 1.5	13 (2.7%)	0 (0%)	
Below 1.5	464 (97%)	77 (100%)	
Missing	8	2	
Platelet Count (10^3^ Platelets/µL)	69 (56 and 124)	50 (33 and 138)	<0.001
Platelet Count, Dichotomized (10^3^ Platelets/µL)			<0.001
At Least 50	454 (94%)	40 (51%)	
Below 50	31 (6.4%)	39 (49%)	
Occurrence of Complication	8 (1.6%)	1 (1.3%)	>0.999
Occurrence of Traumatic Tap	128 (27%)	7 (9.1%)	<0.001
Missing	15	2	
Time to Completion (Hours)	24 (18 and 30)	6 (3 and 21)	<0.001
Occurrence of Platelet Transfusion	261 (54%)	48 (61%)	0.250

The procedure service had an average platelet count of 50,000 platelets/µL in comparison to radiology's 69,000 platelets/µL (p < 0.001). Both teams had a low complication rate, 1.3% for the MPS and 1.6% for radiology, with no statistically significant difference between groups (Table 1). Concordance was 100% between the two attending physicians' interpretations of whether radiologic findings were a possible complication of the procedure. The eight complications in the radiology group included three CSF leaks, one epidural hematoma involving the lumbosacral spinal canal, two subarachnoid hemorrhages of the lumbosacral spinal canal, one case of the development of a cystic structure with adjacent subarachnoid blood, and one small posterior subcutaneous hematoma. The one documented complication from the MPS group was MRI evidence of intrathecal blood products. None of the patients required blood patches.

Despite having lower baseline platelet counts, procedures performed by MPS appear to have a 71% decrease in traumatic tap occurrence compared to those performed by radiology (odds ratio {OR} = 0.29; p = 0.002). This association appears to strengthen after adjusting for platelet count (OR = 0.25; p = 0.001) (Table 2).

**Table 2:** The impact of treating team (medical procedure service versus radiology), platelet count over or below the 50,000 platelets/µL threshold, and treating team adjusted for platelet count on the odds of traumatic tap and completion times for intrathecal chemotherapy. GMR = geometric mean ratios from log-transformed completion times. Values > 1 indicate longer completion times; values < 1 indicate shorter times. All models fitted using generalized estimating equations (GEE) with exchangeable correlation. OR, odds ratio; CI, confidence interval

	Model 1: Treatment Team Medical Procedure Service (Reference: Radiology)		Model 2: Platelet Count Below 50 × 10^3^/µL (Reference: At Least 50 × 10^3^/µL)		Model 3: Treatment Team Medical Procedure Service (Reference: Radiology) Adjusted for Platelet Count	
	OR (95% CI)	p	OR (95% CI)	p	OR (95% CI)	p
Outcome: Any Traumatic Tap (n = 547)	0.29 (0.13, 0.62)	0.002	0.88 (0.47, 1.67)	0.704	0.25 (0.11, 0.57)	0.001
	GMR (95% CI)	p	GMR (95% CI)	p	GMR (95% CI)	p
Outcome: Time to Completion (n = 564)	0.41 (0.32, 0.52)	<0.001	0.53 (0.41, 0.70)	<0.001	0.45 (0.34, 0.59)	<0.001

Procedures performed by MPS appeared to have quicker (59% decrease) time to completion (geometric mean ratio {GMR} = 0.41; p < 0.001) as compared to procedures performed by radiology, and this association held (55% decrease) after controlling for patient platelet count (GMR = 0.45; p < 0.001).

While procedures performed by MPS initially appeared to have higher rates of platelet transfusion as compared to procedures performed by radiology (incidence rate ratio {IRR} = 1.46; p = 0.008), this association was no longer statistically significant after adjusting for platelet count (IRR = 0.85; p = 0.351). Meanwhile, a platelet count below 50,000 is strongly associated with the increased incidence of platelet transfusion (IRR = 1.93; p < 0.001) (Table 3).

**Table 3:** The association of treating team (medical procedure service or radiology), platelet count over or below the 50,000 platelets/µL threshold, and treating team adjusted for platelet count on the incidence rate ratio of platelets transfused. Values > 1 indicate higher transfusion rates; values < 1 indicate lower rates. All models fitted using zero-inflated regression models with a negative binomial logit link. IRR, incidence rate ratio; CI, confidence interval

	Model 1: Treatment Team Medical Procedure Service (Reference: Radiology)		Model 2: Platelet Count Below 50 × 10^3^/µL (Reference: At Least 50 × 10^3^/µL)		Model 3: Treatment Team Medical Procedure Service (Reference: Radiology) Adjusted for Platelet Count	
Outcome: Number of Platelet Transfusions (n = 564)	IRR (95% CI)	p	IRR (95% CI)	p	IRR (95% CI)	p
	1.46 (1.10, 1.93)	0.008	1.93 (1.48, 2.52)	<0.001	0.85 (0.59, 1.20)	0.351

## Discussion

Our study delved into a variety of facets related to receiving intrathecal chemotherapy in the hospital, including timeliness, transfusions, and complications. One of the more striking differences between the radiology and MPS groups was the time from an order for intrathecal chemotherapy being placed to the procedure. MPS teams completed requests for intrathecal chemotherapy more quickly than radiology (six hours versus 24 hours; p < 0.001). During the study timeframe, the MPS was available on some weekends, while radiology only performed these procedures during business hours, excluding weekends and holidays. Another factor that likely contributed to expedited care is the ability to administer intrathecal chemotherapy bedside with the assistance of ultrasound, rather than in a radiology suite with fluoroscopy capabilities. MPS teams are therefore able to reduce a patient's radiation exposure, as well as decrease demand on technologically advanced fluoroscopy units, which can be better utilized for more advanced procedures. Additionally, having a lower cutoff for platelet transfusions likely allowed the MPS to minimize delays in chemotherapy in patients with refractory thrombocytopenia.

The finding of MPS facilitating timely care is supported by studies of other procedures done by MPS teams, such as paracentesis, in which the group that underwent paracentesis by interventional radiology had a 27% longer length of stay (LOS) and 40% longer admission to paracentesis time when compared to the MPS team [21]. Decreasing the time to procedure correlates with decreased LOS and, by extrapolation, the cost of hospitalization and resource utilization. One such example has been a push for the reduction of LOS in patients with community-acquired pneumonia, wherein a reduction of LOS by one half day in this population was correlated with estimated savings of $457-$846 per episode or $500-$900 million annually [22]. This cost savings stands to be even more significant in patients with cancer, in which the average hospitalization costs were $24,653 per admission [23].

Only 5% of patients who underwent intrathecal chemotherapy by our MPS teams needed to be converted to a radiology procedure. Previous studies at other hospitals have reported that their MPS teams' success rate at LPs was 81.3%. In the study by Short et al., patients had a 7.8% minor and 0.1% major complication rate, with 15% of LPs being referred to radiology [9]. In our study, the complication rate was low in both groups despite having a sicker patient population with more thrombocytopenia than the average admitted patients who receive diagnostic LPs. While our study found similar rates of major complications in both study groups (1.3% for MPS and 1.6% for radiology, p > 0.999), we did find that patients who underwent LP with the MPS team had statistically significant lower odds of having a traumatic tap as compared to radiology (OR = 0.29; p = 0.002). Furthermore, since this association appeared to strengthen after adjusting for platelet count, this suggests that practice differences between physician teams seem to drive the differences in traumatic tap occurrences.

The subject of lowering the platelet threshold from the currently accepted 50 × 10^3 ^cells/µL for LP has been heavily debated in the literature. Several studies have identified no significant differences in LP-associated complications or adverse events in patients with hematologic malignancies with platelet counts above or below 50 × 10^9^/L [18,24]. Specifically, one study found that the risk of developing a spinal bleed following an LP was 1.496% in patients with platelets below 50 × 10^9^/L as compared to 1.09% in those above that threshold, a difference that was not statistically significant. This study did, however, find significantly increased odds of receiving a blood patch following an LP in the low-platelet group [25]. Our data demonstrated a low overall complication rate without the need for blood patches in both patient groups. Since the complication rate was very low in our study, further statistical analysis of this patient population and their association with platelet counts yielded very broad confidence intervals for which meaningful inferences could not be extrapolated. We also did not observe an association between platelet count and the rate of traumatic taps. Previous studies are mixed in regard to the association of platelet counts with rates of traumatic tap, with a few studies deeming thrombocytopenia a risk factor [18,26,27], while some found the change to be statistically insignificant [28-30]. Given this conflicting data, a discussion of risks and benefits in conducting procedures at lower platelet thresholds is warranted in these patients, given their relatively low absolute risk of complications.

Blood products are a finite resource that deserve conservation efforts. The MPS at our institution takes a holistic approach to transfusions for patients with hematologic malignancies when the platelets are 20,000-49,000. The proceduralist physician weighs the benefits, indications, patient factors, and how refractory to transfusions the patients have been in the past to determine the best timing of the procedure. Radiology, on the other hand, adheres to a stricter regimen of 50,000 platelets/µL for the vast majority of their patients. In the unadjusted data, the MPS received more units of platelets (OR = 1.46; p = 0.008). However, this association was no longer present when adjusting for patients' platelet counts. Patients with platelet counts of less than 50,000 were proportionally more likely to undergo LPs by the MPS team (43% of patients in the MPS group as opposed to 5.3% in the radiology group) and twice as likely to receive a platelet transfusion (IRR = 1.93; p < 0.001). As this study was performed early in the expansion of the MPS to include intrathecal chemotherapy, many of the patients who were sent to the MPS were patients for whom the primary team was unable to obtain a platelet value of 50,000 after repeated transfusions. Patients with refractory thrombocytopenia may therefore have been self-selected into the MPS group.

While our study has many strengths, particularly the identification of potential complications up to seven days post-procedure, we also have several limitations. First, this is a retrospective study and a single-institution experience. Though many patients remained admitted during our follow-up period, those who were discharged may have presented to other hospitals for the management of their complications or may have had a delayed diagnosis of complications greater than seven days after the procedure. Furthermore, our study was not randomized as radiology or the MPS were consulted by the primary team based on their own judgements. There may have been a perception that more anatomically difficult patients should be sent to radiology. Patients with lower or refractory platelet counts were often referred to MPS teams, as candidacy for the procedure was determined by the clinicians' judgement of the full clinical picture, rather than strict requirements for platelet counts. Patient channeling may have thus been a significant confounder for our results. CSF RBC count was often analyzed in tubes 3 or 4; however, due to the retrospective nature of this study, no protocols existed to standardize the analysis of CSF, and some samples were analyzed in other tubes per the clinician's judgement and orders. The time to procedure may also have been confounded by the availability of teams, since MPS teams were available during weekends whereas radiology was not; however, we would also argue that this is a strength inherent to MPS in that hospitalists are available throughout the weekend.

## Conclusions

Our study suggests that a hospitalist-run MPS can substantially expedite patient care in a vulnerable oncology population with a low complication rate, despite conducting the procedures with a higher proportion of patients with thrombocytopenia. We can reasonably infer that this has the potential to lower costs associated with the procedure, given that it can be done more rapidly and at the bedside; however, formal cost analysis studies are warranted to determine the true fiscal impact of the MPS teams. Furthermore, while our findings indicate that MPS teams can decrease the time to procedure, the effect on total hospital LOS warrants further investigation. Given the low overall complication rate in both our study groups and the lack of association between platelet count and traumatic taps, we would advocate for individualizing decisions regarding LPs in patients with refractory thrombocytopenia and using shared decision-making with patients, given the potential benefits of timely chemotherapy for longitudinal outcomes.

## Appendices

Table 4 shows the statistics for individual patients.

**Table 4:** Statistics for each individual patient, accounting for repeat procedures. Baseline statistics are included to define the incidence or variable at the first intrathecal chemotherapy administration. ^1^Median (Q1 and Q3); n (%). ^2^Wilcoxon rank sum test; Pearson's chi-squared test; Fisher's exact test.

Variable	Radiology (N = 188)^1^	Medical Procedure Service (N = 40)^1^	P-value^2^
Number of Visits	2 (1 and 3)	1 (1 and 2)	0.077
Sex			0.922
Female	83 (44%)	18 (45%)	
Male	105 (56%)	22 (55%)	
Baseline Age (Years)	52 (35 and 66)	40 (28 and 67)	0.144
Median Body Mass Index	27 (23 and 31)	27 (24 and 32)	0.870
Missing	6	1	
Median International Normalized Ratio (INR)	1.10 (1.10 and 1.20)	1.10 (1.00 and 1.20)	0.018
Missing	2	1	
Baseline Platelet Count (10^3^ Platelets/µL)	69 (58 and 113)	65 (35 and 155)	0.040
Median Platelet Count (10^3 ^Platelets/µL)	71 (59 and 118)	57 (36 and 146)	0.015
Baseline Platelet Count, Dichotomized (10^3^ Platelets/µL)			<0.001
At Least 50	178 (95%)	23 (58%)	
Below 50	10 (5.3%)	17 (43%)	
Any Occurrence of Complication Within the Patient	8 (4.3%)	1 (2.5%)	>0.999
Baseline Occurrence of Complication	2 (1.1%)	1 (2.5%)	0.441
Any Occurrence of Traumatic Tap Within the Patient	76 (41%)	7 (18%)	0.005
Missing	3	0	
Baseline Occurrence of Traumatic Tap	44 (24%)	2 (5.0%)	0.007
Missing	5	0	
Baseline Time to Completion (Hours)	24 (19 and 37)	5 (2 and 20)	<0.001
Median Time to Completion (Hours)	24 (19 and 30)	7 (3 and 20)	<0.001
Any Occurrence of Platelet Transfusion Within the Patient	124 (66%)	25 (63%)	0.676
Baseline Number of Platelet Transfusions	1 (0 and 2)	1 (0 and 3)	0.233
Median Number of Platelet Transfusions	1 (0 and 2)	1.75 (0 and 3)	0.120
